# Predicting Diagnostic Gene Biomarkers for Non-Small-Cell Lung Cancer

**DOI:** 10.1155/2016/3952494

**Published:** 2016-07-31

**Authors:** Bin Liang, Yang Shao, Fei Long, Shu-Juan Jiang

**Affiliations:** Department of Respiratory Medicine, Shandong Provincial Hospital Affiliated to Shandong University, Jinan, Shandong 250021, China

## Abstract

Lung cancer is the primary reason for death due to cancer worldwide, and non-small-cell lung cancer (NSCLC) is the most common subtype of lung cancer. Most patients die from complications of NSCLC due to poor diagnosis. In this paper, we aimed to predict gene biomarkers that may be of use for diagnosis of NSCLC by integrating differential gene expression analysis with functional association network analysis. We first constructed an NSCLC-specific functional association network by combining gene expression correlation with functional association. Then, we applied a network partition algorithm to divide the network into gene modules and identify the most NSCLC-specific gene modules based on their differential expression pattern in between normal and NSCLC samples. Finally, from these modules, we identified genes that exhibited the most impact on the expression of their functionally associated genes in between normal and NSCLC samples and predicted them as NSCLC biomarkers. Literature review of the top predicted gene biomarkers suggested that most of them were already considered critical for development of NSCLC.

## 1. Introduction 

Lung cancer is not only the most common cancer in the world, but also the primary reason for death due to cancer [1]. At present, the number of Chinese patients who were newly diagnosed with lung cancer is about 500 thousand each year, and the number is expected to reach one million by 2025 [2]. Non-small-cell lung cancer (NSCLC) is the most common subtype of lung cancer, and NSCLC patients account for about 80% to 85% of all lung cancer incidences [3]. The most common types of NSCLC are squamous cell carcinoma, large cell carcinoma, and adenocarcinoma [4]. Adenocarcinoma is currently the most common type of lung cancer in “never smokers” (lifelong nonsmokers) [5]. Large cell lung carcinoma (LCLC) is a heterogeneous group of undifferentiated malignant neoplasms originating from transformed epithelial cells in the lung [3]. Currently, the most often used treatments for NSCLC are surgery, radiotherapy, and chemotherapy, in which the complete surgical resection is the most effective method [4]. Despite the improvements of surgical techniques and instruments in recent years, the overall 5-year survival rate for NSCLC patients after surgery is from 15% to 45% [6]. On the other hand, most patients that were diagnosed with NSCLC were already in the unresectable stage of IIIB or IV and the option of surgery treatment was not feasible, which is the main reason why the overall 5-year survival rate of NSCLC patients is only 12%. However, the overall 5-year survival rate can be increased to 50% if patients can be diagnosed in an early stage [6]. Currently, the diagnosis of NSCLC mainly depends on X-ray, CT, sputum cytology, fiber bronchoscopy, and cellular pathology. However, X-ray is not sensitive to small lesions and covert lesions; sputum cytology only works on lung cancer originated in the center of airways and has a low detection rate; fiber bronchoscopy is not suitable for early diagnosis due to its invasiveness; cellular pathology cannot precisely determine malignance stages [7]. As such, identifying molecular biomarkers for early diagnosis of NSCLC is urgently needed.

During the development of cancer, tumor cells undergo significant alterations at both genetic and molecular levels, which are accompanied by significant changes in gene expression [8]. Genes with significant expression change during tumor cell development can therefore be used as biomarkers for early diagnosis. For example, Yang et al. conducted a meta-analysis on high-throughput gene expression data of lung cancer and identified tumor necrosis factor-*α* (TNF-*α*) that predicts prognosis of relapse survival for lung cancer [9]. Spinola et al. found that the expression of* PDCD5* was significantly lower in lung cancer patients and suggested that it could be used as a potential molecular marker for predicting the diagnosis of lung cancer [10]. In another example, the expression of* RRM1* encoding ribonucleotide reductase was found to be associated with the response to gemcitabine chemotherapy, making it a prognostic marker for lung cancer [11, 12]. However, identifying gene biomarkers based only on their expression change is not a reliable approach, as the results may be significantly biased by expression noise [13]. On the other hand, since cancer development is a complicated process involving interplay between many genes concordantly, it is necessary to take into consideration the complicated relationships between genes when identifying gene biomarkers of diagnostic use. Thus, a number of methods have been developed to integrate network or pathway information for discovering disease genes [14, 15].

Recently, Long et al. developed an integrated approach for predicting biomarkers that distinguish NSCLC from small-cell lung cancer (SCLC) [16]. In their approach, they first constructed a lung cancer-specific functional network by integrating gene coexpression relationships identified in NSCLC, SCLC, and normal lung tissues with gene functional associations obtained from the STRING network [17]. Then, they applied a network partition algorithm to identify gene modules from the lung cancer-specific network and selected the gene modules with distinctive gene expression pattern in between NSCLC and SCLC. Finally, from the selected gene modules, they identified those genes whose functionally associated partners exhibit significant gene expression alterations in between NSCLC and SCLC and predicted them as candidate biomarkers. Inspired by their approach, in this study, we aimed to predict gene biomarkers that distinguish NSCLC from normal lung tissues and therefore might be of use for diagnosis of NSCLC.

## 2. Materials and Methods

### 2.1. Dataset Collection, Processing, and Construction of Lung Cancer-Specific Functional Association Network

In Long et al.'s study [16], they obtained three gene expression datasets for NSCLC and SCLC from Gene Expression Omnibus (GEO) database of NCBI (http://www.ncbi.nlm.nih.gov/geo/) by following these criteria: samples must be from untreated human tissue samples and the sample size should be greater than 100. Since one of the expression datasets was SCLC, here we only used two expression datasets: normal lung tissue dataset (GSE23546) and NSCLC dataset (GSE41271) that include 1349 and 275 samples, respectively. We followed Long et al. [16] to process the normal and NSCLC datasets separately and to construct the lung cancer-specific functional association network. Briefly, after normalizing gene expression profiles of normal and NSCLC samples separately, we calculated the Pearson Correlation Coefficient (PCC) of gene expression levels for every gene pair in the two datasets separately and selected the top 1% and the bottom 1% of gene pairs ranked by PCC, representing a strong relationship of positive and negative coexpression, respectively. The human STRING network (version 9.1) [18, 19] was downloaded from http://string.embl.de/, which consists of 19,038 genes and 2,271,610 weighted edges. Next, we filtered the STRING network by requiring the functional association scores between two genes to be greater than 500. Finally, we mapped the coexpression gene pairs identified in both normal and NSCLC datasets to the filtered STRING network and obtained a lung cancer-specific functional association network. In this network, any two linked genes not only are strongly functionally associated, but also are either positively or negatively coexpressed in normal or NSCLC samples.

### 2.2. Network Partition and the Identification of NSCLC-Specific Gene Modules

Again, following what was already described, we applied a network partition algorithm named iNP [28] to divide the lung cancer-specific network into gene modules. In order to guarantee each gene module including several genes, we also set the number of genes inside a module to be greater than 4 so that we can get some stable modules. From these gene modules, we identified the NSCLC-specific gene modules by inspecting their differential expression pattern in between NSCLC and normal samples. Briefly, for each module, we recorded the median expression value in each sample as the representative expression value for the module; then, we applied *t*-test in *R* to determine the significance of the genes' differential expression in between normal and NSCLC samples. Following Long et al. [16], we used the Benjamini-Hochberg false discovery rate (FDR) [29, 30] to conduct multiple test correction and chose the cutoff to adjust *p* value at 0.01 and also required log (fold change) ≥2 to select candidate gene modules. These gene modules were considered NSCLC-specific gene modules. To quantify the specificity of each selected module to NSCLC, for each module, we plotted a receiver operating characteristic (ROC) curve by applying the modules' median gene expression value to distinguish NSCLC from normal cases. The area under the ROC curve (AUC) could be greater or smaller than 0.5, corresponding to up- or downregulated gene expression patterns in NSCLC in comparison to their expression levels in normal case. The farther the AUC is from 0.5, the more specific the expression of a module is to NSCLC.

### 2.3. Gene Set Enrichment Analysis

Gene set enrichment analysis was performed by Fisher test in *R*. Gene set annotations include gene ontology (GO) biological process terms [31] and gene sets from molecular signatures database (MSigDB) [32]. Multiple test correction was conducted by FDR in *R*.

### 2.4. Scoring Genes for Candidate Gene Biomarkers of NSCLC

Given a selected gene module, we followed Long et al. [16] to score each gene inside the module for the genes' usefulness as NSCLC biomarker. Briefly, there were two component scores: the cancer-specificity score determined by the AUC_ROC of the module the gene belongs to minus 0.5 and the coexpression change score of the gene. To obtain the coexpression change score, all genes connecting to the target gene were first recorded. Then, for each connection, a coexpression status difference score was computed by using the coexpression status of the two genes in NSCLC minus their coexpression status in normal case. The coexpression status can take three scores: 1, −1, and 0, indicating positive correlation, negative correlation, and others, respectively. Then, the absolute coexpression status difference was averaged across all connections to derive the coexpression change score, which indicated the ability of the gene to impact the expression pattern of its functionally associated genes. The two component scores were then multiplied, and a positive score indicated the gene tended to be upregulated in NSCLC, or vice versa.

## 3. Result

### 3.1. Construction of an NSCLC-Specific Functional Association Network

**Figure 1:**
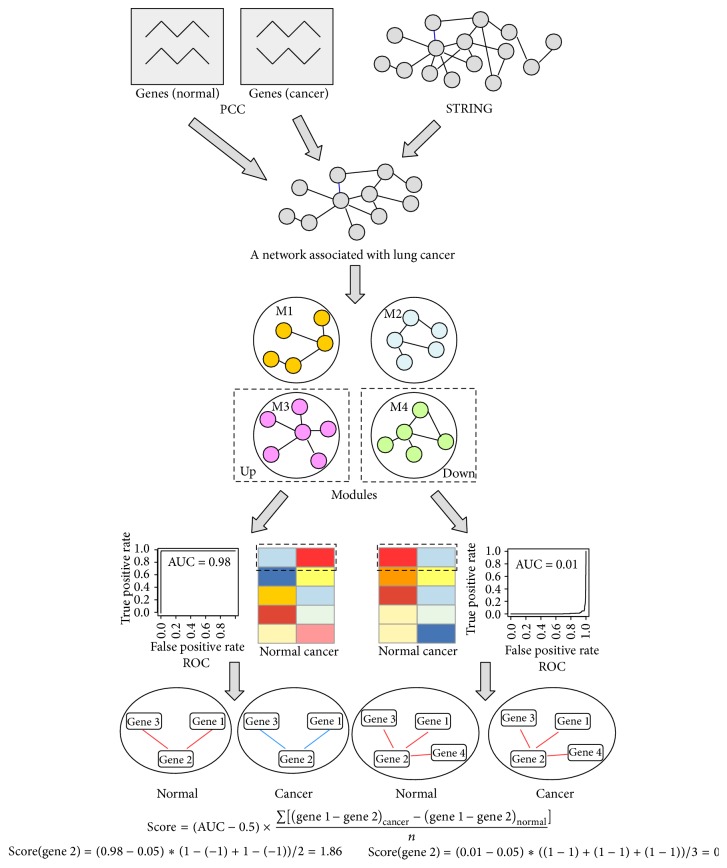
The pipeline for predicting diagnostic gene biomarkers of NSCLC. In the bottom of the figure, the color of the line connecting gene 2 and the other genes indicates the coexpression status between gene 2 and the other genes, with red color corresponding to positive coexpression and blue color corresponding to negative coexpression. For details about the pipeline, refer to the Materials and Methods.

The pipeline for predicting NSCLC diagnostic gene biomarkers is shown in Figure 1. The rationale of the methodology behind the pipeline was that, given a gene module in which genes not only were functionally associated, but also had correlated expression patterns, if the module's overall expression pattern (the median expression value of genes inside a module) was significantly different in between cancer and normal samples, then this module could be considered a cancer-specific gene module; within this cancer-specific gene module, if a gene's expression correlations with its functionally associated partners were significantly altered from normal to cancer samples, then this gene's expression might be critical to cancer development and was then considered a potential biomarker for diagnostic use. For details about the methodology, please refer to Long et al. [16]. Briefly speaking, the pipeline consists of the following three steps: construction of a disease-specific functional association network by integrating gene coexpression with functional association, identification of disease-specific gene modules, and prediction of gene biomarkers. Following the pipeline, we have constructed an NSCLC-specific functional association network by integrating gene coexpression information obtained from both lung normal and NSCLC datasets with the filtered functional association extracted from the STRING network (see Materials and Methods for details). This network is a binary network consisting of 4,452 genes and 13,831 edges.

### 3.2. Identification of NSCLC-Specific Gene Modules

The NSCLC-specific network was partitioned into 637 gene modules by using a network partition algorithm named iNP. There are 254 gene modules with the cutoff of including more than 4 genes. To identify NSCLC-specific gene modules, following Long et al. [16], we used the median gene expression value of the genes inside the module to represent the module's expression value in normal or NSCLC dataset and then inspected the differential expression pattern of each module between normal and NSCLC samples (see Materials and Methods for details). We found 11 gene modules whose expression was significantly different in between normal and NSCLC samples. There are 2 upregulated modules and 9 downregulated modules among these differentially expressed modules. The representative expression values of each of the 11 modules were also used to plot ROC curves to discriminate NSCLC samples from normal samples, from which the area under curve (AUC) was computed. Here, we labeled cancer samples with “1” and normal samples with “0.” Then, by sorting the gene expression values of a module in each sample from high to low values, we computed the true positive rate (number of samples with label of “1” above the value/number of all samples with label “1”) and false positive rate (number of samples with label of “0” above the value/number of all samples with label “0”) at each expression value. Finally, a ROC curve was obtained by plotting the true positive rate against the false positive rate. If the expression values of a module in cancer samples were similar to that in normal samples, then the AUC of the ROC curve would be close to 0.5. If a module typically had higher gene expression values in cancer samples (upregulated in cancer samples), then its AUC would be greater than 0.5; otherwise (downregulated) it would be smaller than 0.5. The farther the AUC of a ROC from 0.5, the more significant the difference that we would observe in between the gene expression values of cancer and normal samples. In other words, the AUC of a ROC curve quantitatively determines the specificity of a module to NSCLC. Because AUC_ROC measures the expression alteration of a group of genes that are coexpressed, it is more robust than the expression alteration of individual genes and should be more appropriate to be used to represent the expression change of a given gene of interest. Here, we found 2 modules with AUC value greater than 0.9 (significantly upregulated in NSCLC) and 9 with AUC value smaller than 0.1 (significantly downregulated) (Table 1).

To validate the specificity of these modules to lung cancer, we performed function enrichment analysis for genes inside each module and showed the most significant function for each module in Table 1. It could be easily seen that the enriched functions of selected modules were significantly associated with cancer's development. In the upregulated gene modules, M44 has 5 genes with an AUC of 0.996; the top enriched function of this module was protein targeting to membrane (*p* value: 1.68*E* − 04). Recently, Li and Perez-Soler [33] found that skin toxicity is related to inhibition of epidermal growth factor receptor (EGFR), a target for NSCLC treatment. Another example of upregulated modules is M394 that consisted of 15 genes with an AUC of 0.909. The top enriched function was epidermis development with a *p* value of 1.80*E* − 08. Tian et al.'s [34] research result showed that an important gene in epidermis development,* DHHC*, encoding palmitoyltransferase catalyzes S-palmitoylation by targeting on the cell membrane, and siRNA targeting this gene was able to inhibit the growth of NSCLC cell lines. There were nine NSCLC-specific gene modules that were downregulated. Among these modules, M315 consisted of 9 genes and had an AUC of 0.0913. The top enriched function was cell chemotaxis (8.50*E* − 07), which was found to promote the development of NSCLC [35]. In another example, M349 consisted of seven genes with an AUC of 0.0091. All these seven genes were associated with the function of BMP2 targeting with a *p* value of 0.00159. Gautschi et al. [36] found that Id1 plays an important role in Src-mediated tumor cell invasion, and BMP2 could induce the expression of Id1, suggesting that BMP2 targeting might be important for cancer development. Therefore, functional enrichment results validated the specificity of the partitioned gene modules to NSCLC.

### 3.3. Predicting Diagnostic Gene Biomarkers for NSCLC

After identifying NSCLC-specific gene modules, we aimed to determine the genes inside an NSCLC-specific module that have the best discriminating power to distinguish NSCLC from normal tissues. Following the strategy described by Long et al. [16], we computed the module's specificity to NSCLC, which is AUC 0.5. Then, for each gene inside a cancer-specific module, we computed a coexpression change score to indicate its potential impact on the alteration of the coexpression pattern of its functionally associated partner genes. For details about the score, please refer to the Materials and Methods. The final score can be either positive or negative, indicating that the corresponding gene is either upregulated or downregulated in NSCLC, respectively.

**Figure 2:**
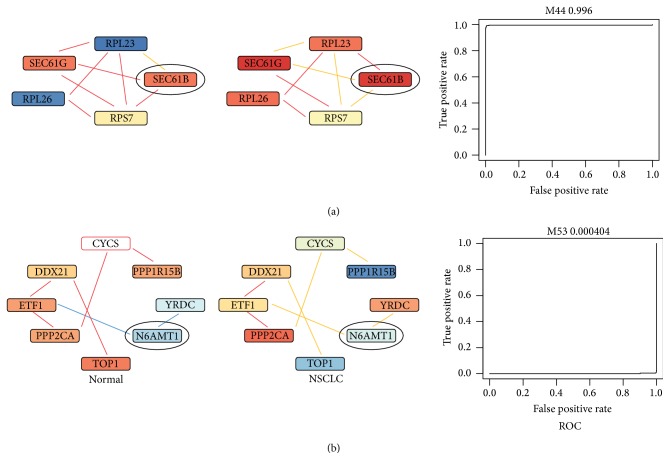
The predicted gene biomarkers for NSCLC. (a) shows an example of upregulated gene biomarker—*SEC61B.* Its coexpression patterns with the functionally associated genes in normal and NSCLC were shown separately. Line colors of red, blue, and orange indicated the positive, negative, and random coexpression. (b) is similar to (a), except that the example is a downregulated gene biomarker—*N6AMT1*. The color of each gene represented its expression level. The ROC curves to the right were based on the corresponding gene modules of* SEC61B* and* N6AMT1*.

We obtained 59 genes with nonzero scores. Among them, 11 were upregulated and 48 were downregulated. In Table 2, we listed the top 10 upregulated and top 10 downregulated genes and considered them as potential biomarkers for NSCLC.* SEC61B* was ranked the top among the upregulated genes. It was functionally associated with three genes (*SEC61G*,* RPL23*, and* RPS7*) in gene module M44 that was significantly associated with NSCLC (the AUC of the ROC curve by using the median gene expression value of M44 to discriminate NSCLC from normal case was 0.996). As shown in Figure 2, the coexpression pattern of* SEC61B* with its functionally associated genes changed significantly from normal to NSCLC samples. For example,* SEC61B* and* RPL23* were not positive or negatively coexpressed in normal case; however, they were significantly positively coexpressed in NSCLC. As for* SEC61G* and* RPS7*, they were both positively coexpressed with* SEC61B* in normal samples; in NSCLC samples, however, they were no longer positively coexpressed with* SEC61B.* The significant changes in the coexpression pattern of genes functionally associated with* SEC61B* in between normal and NSCLC cases indicated that it might be an important gene that could potentially have a large impact on NSCLC's development.* N6AMT1* ranked the top among downregulated gene biomarkers. It had two functionally associated genes* YRDC* and* ETF1* in gene module M53 whose AUC value was 0.0004. Interestingly, the coexpression pattern of both* YRDC* and* ETF1* with* N6AMT1* reversed from normal to NSCLC samples: they were both negatively coexpressed with* N6AMT1* in normal lung samples, while they were both positively coexpressed with* N6AMT1* in NSCLC samples (Figure 2). Thus, it was very likely that the coexpression of* N6AMT1* with its functionally associated genes may play important roles in the development of NSCLC.

### 3.4. Case Reports for the Predicted Gene Biomarkers

We have conducted a thorough literature review on the predicted gene biomarkers. As shown in Table 2, 9 of them were known to be relevant to NSCLC, and the other 11 were known to be relevant to cancer, strongly validating our predictions and also suggesting that they may be of use as diagnostic biomarkers for NSCLC. Below, we presented an example for both upregulated and downregulated gene biomarkers, respectively. One example is* S100P* that encodes a member of small calcium-binding proteins family and is highly expressed in NSCLC.* S100P* played a key role downstream for Keap1-Nrf2 interaction.* Keap1* encodes E3 ligase and is involved in cellular defense response to oxidative stress through an interaction with nuclear factor erythroid-2-related factor 2 (Nrf2). It has been shown that* Keap1* could inhibit tumor metastasis by targeting Nrf2/S100P pathway in NSCLC cells [20]. Thus,* S100P* is of high importance to NSCLC. In another example, FOXA2 is a tumor suppressor and has been suggested to be a new target protein for the treatment of NSCLC [25].

### 3.5. The Validation of Analysis Strategy

Following Long et al. [16], in order to prove the availability and reasonability of the method, we used another NSCLC dataset (GSE10245; detailed information was shown in Long et al. [16]) to repeat the analysis for predicting gene biomarkers. Based on this dataset, we predicted 84 genes biomarkers for NSCLC, with 12 overlapping predictions. The number of overlapped predictions was significantly higher than random guess (<10–4, 10,000 randomizations), proving the robustness of our predictions.

## 4. Discussion

Lung cancer is the primary reason for death due to cancer worldwide, and NSCLC is the most common subtype of lung cancer. In the present study, we predicted 20 gene biomarkers potentially useful for diagnosis of NSCLC. Literature reviews of our predictions revealed that some of them were already reported to be specifically relevant to NSCLC, while the others were relevant to cancer. As such, the predicted gene biomarkers may be of use for further exploitation as diagnostic biomarkers for NSCLC.

The predicted gene markers have the following characteristics. First of all, they are within gene modules in which genes are strongly functionally associated with each other based on the STRING network and have correlated expression in normal or NSCLC samples. In addition, the modules are specific to cancer as measured by the median expression values of genes inside the module. Thirdly, the predicted genes had altered expression correlation with their functionally associated partners in between normal and NSCLC samples. For instance, a gene is positively correlated with its functionally associated partners in normal samples in terms of gene expression, while this pattern significantly altered to negative correlation. This abnormal coexpression pattern alteration thus made the gene potentially important for NSCLC cancer cell development. Thus, the abnormal coexpression pattern alteration could be potentially useful for diagnostic use. Specifically, rather than focusing on the expression value alteration of the predicted gene biomarkers, we ought to consider the expression of both the predicted gene biomarkers and their functionally associated partner genes. For example, we could develop a microchip that integrates not only the predicted gene biomarkers but also their functionally associated partners, and then, by examining the expression of these genes as a whole, we could develop a system to detect the abnormal coexpression alteration for assessing whether a sample is NSCLC. The predicted biomarkers were ranked according to our scoring scheme that considers not only the level of expression alteration, but also the extent of the expression change of the biomarkers' partner genes. Therefore, a gene with higher rank should not only have a more significant expression alteration in cancer, but also have higher impact on the expression of its partner genes. Consequently, this gene should be more easily detected than those genes with lower ranks and should be studied with higher priority.

Given the predicted biomarkers and their partner genes for NSCLS, an immediate application is for clinical diagnostic use of NSCLC. By developing a microchip to measure the expression of these biomarkers and their partner genes, we can collaborate with local hospitals to apply this chip to NSCLC patients and normal people. Then, we can construct a clinical model based on the results from hundreds of patients and normal people. This model will be used to diagnose whether a new patient likely has NSCLC by measuring the expression of the predicted biomarkers and their partners in the chip.

## Figures and Tables

**Table 1:** The 11 cancer-specific gene modules.

Module name	AUC_ROC	The most significantly enriched function (*p* value)
M44	0.996	GO: protein targeting to membrane (0.00110)
M394	0.909	GO: epidermis development (1.80*e* − 08)
M53	0.000404	MSigDB: CTRL_VS_DAY3_LAIV_IFLU_VACCINE_PBMC_UP (0.00670)
M348	0.00113	MSigDB: MIKKELSEN_IPS_LCP_WITH_H3K4ME3 (0.00670)
M350	0.00313	MSigDB: BOSCO_TH1_CYTOTOXIC_MODULE (1.80*e* − 08)
M60	0.00833	MSigDB: MCLACHLAN_DENTAL_CARIES_UP (8.66*e* − 11)
M349	0.0091	MSigDB: BMP2_TARGETS_UP (0.00159)
M264	0.0159	MSigDB: SHEDDEN_LUNG_CANCER_GOOD_SURVIVAL (5.66*e* − 04)
M83	0.0273	MSigDB: BOYLAN_MULTIPLE_MYELOMA (9.10*e* − 04)
M94	0.0284	None
M315	0.0913	GO: cell chemotaxis (8.50*e* − 07)

**Table 2:** Summary of the top 10 upregulated and top 10 downregulated gene biomarkers for NSCLC.

Gene rank	Gene name (upregulated)	Gene name (downregulated)
1	SEC61B	N6AMT1
2	S100P^*∗*^ [20]	CYCS
3	RPL23	YRDC
4	SEC61G	PPP1R15B
5	SPRR2D^*∗*^ [21]	TOP1^*∗*^ [22]
6	RPS7	MMRN2
7	S100A2^*∗*^ [23]	P2RY14^*∗*^ [24]
8	SPRR3	FOXA2^*∗*^ [25]
9	DSG3^*∗*^ [26]	GCA^*∗*^ [27]
10	SPRR1A^*∗*^ [21]	MGAM

*∗* indicates that the gene was relevant to NSCLC directly, with the references shown in parenthesis.
